# Efficacy of Probiotic Compounds in Relieving Constipation and Their Colonization in Gut Microbiota

**DOI:** 10.3390/molecules27030666

**Published:** 2022-01-20

**Authors:** Yuan He, Leilei Zhu, Jialun Chen, Xin Tang, Mingluo Pan, Weiwei Yuan, Hongchao Wang

**Affiliations:** 1 State Key Laboratory of Food Science and Technology, Jiangnan University, Wuxi 214122, China; 6200113031@stu.jiangnan.edu.cn (Y.H.); 7190112086@stu.jiangnan.edu.cn (L.Z.); 6190111135@stu.jiangnan.edu.cn (M.P.); 6180111089@stu.jiangnan.edu.cn (W.Y.); 2 School of Food Science and Technology, Jiangnan University, Wuxi 214122, China; 3 Sirio Pharma Co., Ltd., Shantou 515000, China; dane.chen@siriopharma.com (J.C.); xin.tang@siriopharma.com (X.T.)

**Keywords:** constipation, probiotic compounds, gastrointestinal transit rate, gut microbiota, colonize

## Abstract

A number of studies have confirmed the relationship between constipation and gut microbiota. Additionally, many human and animal experiments have identified probiotics as effectors for the relief of constipation symptoms. In this study, probiotic compounds, including *Lactobacillus acidophilus* LA11-Onlly, *Lacticaseibacillus rhamnosus* LR22, *Limosilactobacillus reuteri* LE16, *Lactiplantibacillus plantarum* LP-Onlly, and *Bifidobacterium animalis* subsp. *lactis* BI516, were administered to mice with loperamide-induced constipation, and the impacts of these strains on constipation-related indicators and gut microbiota were evaluated. The effects of probiotic compounds on constipation relief were associated with various aspects, including gastrointestinal transit rate, number and weight of stools, serum and intestinal gastrointestinal regulatory hormones, and serum cytokines. Some of the probiotic compounds, including *Limosilactobacillus reuteri*, *Lactiplantibacillus plantarum*, and *Lacticaseibacillus rhamnosus*, were found to colonize the intestinal tract. Furthermore, higher dosages promoted the colonization of specific strains. This study yields a new perspective for the clinical use of probiotics to improve constipation symptoms by combining strains with different mechanisms for the alleviation of constipation.

## 1. Introduction

Constipation is a common, burdensome functional bowel disorder in which symptoms of difficult, infrequent, or incomplete defecation predominate [1]. It has a negative effect on quality of life [2]. Approximately 12–30% of people worldwide suffer from constipation [3]. Constipation not only increases the financial burdens of patients, but also increases the social burden of the healthcare system [4]. Some studies have shown that the probability of constipation symptom onset increases beyond the age of 65, and is twice as high in women as in men [5,6]. Despite the range of treatments available, including laxatives and fiber supplements, approximately half of patients are dissatisfied with current management strategies, with the main complaint being limited efficacy [7,8]. Hence, there is an unmet need for alternative treatments for the management of constipation-related symptoms.

The gut is home to trillions of microbes, the composition of which correlates with many disease states. The gut microbiota serves as an endocrine organ, facilitating the production and regulation of various neurotransmitters and hormones. While recent studies have started to unravel the effects of this vast microbial community and its complex metabolic repertoire on intestinal physiology, its true potential remains underexplored [9]. Accumulated evidence shows that some probiotic strains ameliorate functional constipation (FC) via the modulation of specific gastrointestinal peptide pathways [10,11]. In the past decade, research has focused on their effectiveness for treating constipation, possibly mediated through regulation of gut dysmotility by impacting the gut microbiota, with the subsequent release of metabolites such as tryptamine [12] and short-chain fatty acids (SCFAs) during fermentation, which are known to interact with the intestinal barrier, intestinal immune system, and nervous system. Thus, new trends in constipation management have considered probiotic administration as a possible strategy.

Most research has studied the relieving effect of a single strain of probiotics on mice with constipation, and we studied combinations of a variety of probiotics (*Lactobacillus acidophilus* LA11-Onlly, *Lacticaseibacillus rhamnosus* LR22, *Limosilactobacillus reuteri* LE16, *Lactiplantibacillus plantarum* LP-Onlly, *Bifidobacterium animalis* subsp. *lactis* BI516) that can relieve constipation. The aim of this study was to investigate the roles of probiotic compounds in relieving constipation and colonizing the intestine. For this, the effects of probiotic compounds on gastrointestinal (GI) transit rate, serum and intestinal gastrointestinal regulatory hormones, and serum cytokines were assessed in mice with constipation. The effects of probiotic compounds on regulating the gut microbiome and their ability to colonize the intestinal tract were assessed. Overall, this study aids our understanding of the relationship between probiotic compounds and constipation, providing insights into a new intervention strategy for constipation.

## 2. Materials and Methods

### 2.1. Preparation of Probiotic Suspensions

*Lactobacillus* (*L. acidophilus*, *L. rhamnosus*, *L. reuteri*, *L. plantarum*) culture conditions were 37 °C for 16 h. The conditions of *Bifidobacterium* (*Bifidobacterium animalis subsp. lactis*) were anaerobic culturing at 37 °C for 30 h. The two-generation activated strains were placed in 1 L of fresh culture medium; the inoculum amount was 2%. These cultures were placed in the incubator with the corresponding conditions. The obtained bacterial liquid was centrifuged at 6000× *g* for 15 min. After the bacteria were washed twice with saline solution, they were resuspended with a sucrose solution (as a protective solution) and stored at −80 °C for later use. We measured the concentration of the bacterial solution before use. The probiotic compounds were prepared by Sirio Pharma. They contained only compounds of the five strains above.

We mixed the probiotic compounds and saline solution in proportion to make a probiotic suspension for subsequent gavage into mice. We ensured that the concentration of each probiotic reached 2.5 × 10^9^ CFU/mL for the low-dose group, and 2.5 × 10^10^ CFU/mL for the high-dose group.

### 2.2. Animals and Experimental Design

**Figure 1:**
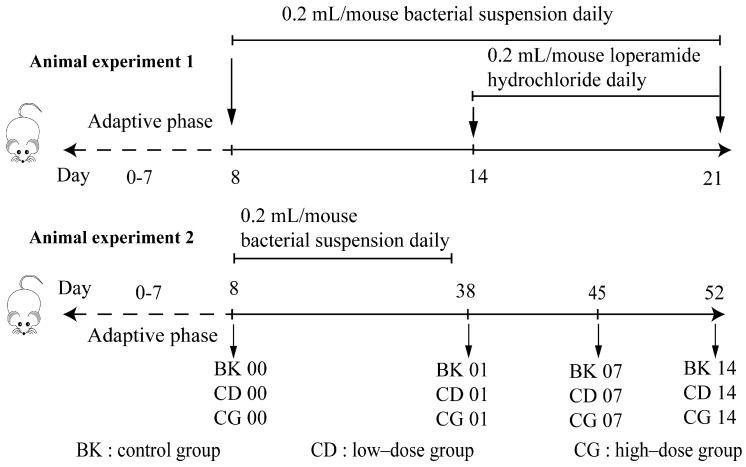
Animal experimental groups. Normal group, BK; low–dose group, CD; high–dose group, CG; the first gavage, 00; the last gavage, 01; one week after stopping gavage, 07; two weeks after stopping gavage, 14.

Six-week-old, male, SPF-grade BALB/c mice were obtained from Shanghai Slack Company. The animal experimental protocol was approved by the Ethics Committee of Jiangnan University (JN.No20190930b0600120) and implemented in accordance with existing EU guidelines (2010/63/EU). The breeding environment was maintained at a temperature of 23 ± 2 °C and relative humidity of 50 ± 10%, with a 12/12 h light/dark cycle. Fifty mice were randomly divided into a normal group, model group, positive group (*Lactobacillus rhamnosus* GG 0.1 g/kg BW bacterial suspension), low-dose group (0.1 g/kg BW bacterial suspension), and high-dose group (1.0 g/kg BW bacterial suspension)—ten mice per group. All animal experiments were performed in strict accordance with the regulations of the Animal Management and Use Committee of Jiangnan University (SYXK 2012-0002). The mice were gavaged daily after 7 days of adaptation to the environment. During days 8–14, mice in the normal and model groups were intragastrically administered 0.2 mL of saline solution, while the other groups were given 0.2 mL of bacterial suspension daily. All mice except for those in the normal were gavaged with loperamide hydrochloride daily (10 mg/kg BW) during days 15–21, while the mice in the normal group were orally administered saline solution daily. Low and high-dose groups were gavaged bacterial suspension continuously; the loperamide hydrochloride treatment was performed 1 h before bacterial suspension gavage daily on days 15–21 (Figure 1). The mice were anesthetized with isoflurane inhalation before pricking an eyeball for collecting blood. Carbon dioxide euthanasia and cervical dislocation were performed after blood collecting.

Thirty mice were randomly divided into a control group (BK), low-dose group (CD) (0.1 g/kg BW bacterial suspension), and high-dose group (CG) (1.0 g/kg BW bacterial suspension)—ten mice per group. During days 8–37, mice were given 0.2 mL of bacterial suspension daily in the CD and CG groups, and the mice in BK were orally administered saline solution (vehicle) daily. Mouse feces were collected in sterile tubes, snap-frozen on day 38, day 45, and day 52, and stored at −80 °C for high-throughput sequencing (Figure 1). The experimental conditions were consistent with those described above. After 52 days of treatment, the mice were sacrificed via carbon dioxide euthanasia and cervical dislocation.

### 2.3. Determination of the Time Taken for First Black Stool

On day 21, the fifty mice were intragastrically administered activated carbon mixed with probiotic suspensions (or saline solution). The mice were then placed into clean, dry cages, and the time when each mouse expelled its first black stool, along with the number and weight of black stools discharged within six hours, were recorded.

### 2.4. Determination of the Gastrointestinal Transit Rate

On day 21, the mice were starved overnight (water was provided). At 8:30 a.m. on day 22, 0.2 mL distilled water was given to mice in the normal group, while all other mice were given 0.2 mL of loperamide hydrochloride solution (10 mg/kg BW). After 30 min, activated carbon was intragastrically administered to all mice. After another 30 min, the mice were sacrificed with carbon dioxide euthanasia and cervical dislocation. The entire small intestine from the pylorus to the caecum was removed, and the total length of the small intestine was measured. The distance to the front edge of the activated carbon was also measured. For each mouse, the GI transit rate was calculated as the percentage of prosthetic advancement of the activated carbon relative to the total length of the small intestine.

### 2.5. Determination of Peptide and Serotonin Factor Concentrations

Mice that had starved for 12 h were intraperitoneally injected with a sodium pentobarbital solution (0.5 mL/10 g BW) and subsequently sacrificed. Serum samples were obtained from animal experiment 1’s blood samples by centrifugation (3000× *g*, 15 min) in which the concentrations of motilin (MTL), substance P (SP), endothelin (ET-1), somatostatin (SS), vasoactive intestinal peptide (VIP), gastrin (GAS), and cytokines (IL-2, IL-4, IL-6, IL-10, IL-12, IL-17) were measured using commercial ELISA kits (Nanjing SenBeiJia Biological Technology Co., Ltd., Nanjing, China) according to the manufacturer’s instructions. The colon tissue was washed with pre-cooled phosphate buffered saline (PBS), the surrounding adipose tissue was removed, and the colon was cut and weighed. The tissue was disrupted using a tissue disrupter in a corresponding volume of PBS (weight to volume ratio of 1:9), and finally centrifuged at 5500× *g* for 10 min. The supernatant was taken to detect the concentrations of gastrointestinal regulatory peptides.

### 2.6. DNA Extraction from Fecal Samples and High-Throughput Sequencing of the Gut Microbiota

Fecal samples were collected in appropriate sterile tubes to explore changes in the gut microbiome. Each fecal sample was frozen immediately at −80 °C to prevent distortion of the bacterial community profile. Each fecal sample (200 mg) was subjected to DNA extraction using the FastDNA Spin Kit for Feces (MP Biomedicals, Solon, OH, USA). The V3–V4 region was amplified using polymerase chain reaction (PCR), and the PCR primers were forward primer 5′-CCTAYGGGRBGCASCAG-3′ and reverse primer 5′-GGACTACNNGGGTATCTAAT-3′ (Supplementary Table S1). After PCR, an agarose gel was cut for purification using the DNA Gel/PCR Purification Miniprep Kit (BW-DC3511, Beiwo Meditech Co., Ltd., Hangzhou, China) according to the manufacturer’s instructions. The DNA was further targeted for amplification in order to quantify the species of *Lactobacillus* and *Bifidobacterium* in feces: *Bifidobacterium*-specific primers (Bif-GroelF(5’-TCCGATTACGAYCGYGAGAAGCT-3’) and Bif-GroelR (5’-CSGCYTCGGTSGTCAGGAACAG3’)) and *Lactobacillus*-specific primers (Lac-GroelF (5’-GCYGGTGCWAACCCNGTTGG-3’) and Lac-GroelR (5’-AANGTNCCVCGVATCTTGTT-3’)) were used to amplify specific fragments of the Groel genes of *Bifidobacterium* and *Lactobacillus*, respectively. A specific adapter was used for subsequent sequencing on the MiSeq PE300 platform (Illumina, Santiago, CA, USA).

### 2.7. Gut Microbiome Analysis

Paired-end sequencing reads were merged using QIIME2. The reads were demultiplexed and quality filtered using Quantitative Insights into Microbial Ecology software (QIIME2) [13]. To get good operational taxonomic unit sequences (OTUs), low-quality reads should be discarded because they often cause spurious OTUs. The OTUs were chosen to ensure 97% identity. The latest Silva 16s rRNA v132 database and the Groel gene sequence database cpnDB were used to produce the most precise species composition results. Taxonomy assignment of V3–V4 was performed within QIIME2. Taxonomy assignments of *Lactobacillus* and *Bifidobacterium* proceeded as in the previous article [14,15]. To avoid bias due to different sequencing depths, the OTU tables were rarefied to 10,000 sequences per sample for computing alpha-diversity metrics (Pielou evenness, Faith_pd, Observed–otus and Shannon) within QIIME2. Beta-diversity was calculated with the Bray–Curtis by the method of non-metric multidimensional scaling (NMDS) and principal component analysis (PCoA) [16]. We analyzed the differences between the groups of bacteria through the method of liner discriminant analysis effect size (LEfSe) [17]. To construct the percentage accumulation map of the microbiota, we first removed genera with relative abundances of less than 0.01%.

### 2.8. Evaluation of Strain Colonization Characteristics

The feces were collected before the test: one day after the last gavage, one week after the last gavage, and two weeks after the last gavage for the CD, CG, and BK groups. A known number of artificially synthesized spike plasmids were added to the sample. Then we performed bioinformatic analysis after sequencing for absolute quantitative analysis. That way, we detected the absolute abundances of bacteria in feces collected at different time points.

### 2.9. Statistical Analysis

The statistical analyses were processed with GraphPad Prism 8 (La Jolla, CA, USA) and IBM SPSS Statistics 20 (SPSS Inc., Chicago, IL, USA). All data are expressed as mean ± standard error of the mean. Significant differences between groups were analyzed using one-way analysis of variance (ANOVA); *p* < 0.05 was considered statistically significant (confidence interval > 95%). Gut microbiota diversity and phylum taxon abundance were compared using the Wilcoxon rank-sum test in R statistical software package 4.1.0 (https://www.r-project.org/, accessed on 1 December 2021). The statistical analyses of the Gut microbiota were processed with R statistical software package 4.1.0.

## 3. Results

### 3.1. Probiotic Compounds Significantly Regulate Intestinal Health in Mice with Constipation

**Figure 2:**
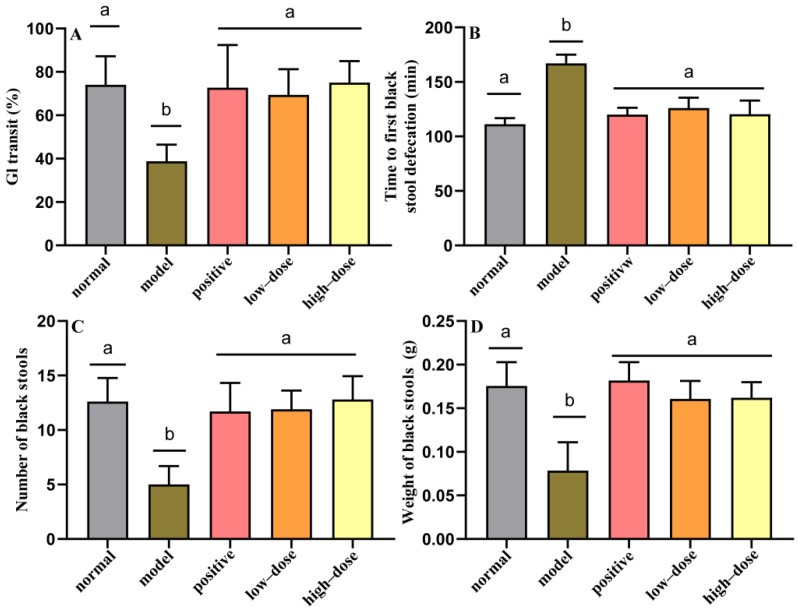
Effects of probiotic compounds on defecation in a mouse model of constipation. (**A**) Gastrointestinal transit rates. (**B**) Times to first black stool defecation. (**C**) Number of black stools. (**D**) Weight of black stools. Data are means with SEM. a–d: Different letters indicate significant differences (*p* < 0.05), and the same letters or no letters indicate no significant difference. Same as below.

The GI transit rate reflects the dynamics of the small intestine and may thus reflect the effects of probiotic compounds on intestinal motility. A higher GI transit rate indicates a shorter residence of chyme in the small intestine and greater small intestinal motility. Loperamide challenge led to a low GI transit rate in the model group, indicating that the constipation model was successfully established. After the intragastric administration of probiotic compounds, mice with constipation in low and high-dose groups exhibited significantly improved GI transit rates (*p* < 0.05) (Figure 2A). This observation suggests that treatment with probiotic compounds can effectively improve intestinal motility and accelerate the movement and exit of chyme via the small intestine. There was no significant difference in GI transit rate between the low and high-dose group.

The time till the first black stool defecation represents motility throughout the intestine. Specifically, a shorter time till the first black stool defecation indicates more rapid whole intestinal tract motility and a stronger intestinal transport capacity. After 7 days of loperamide challenge, the time till first black stool defecation was significantly longer in the model group than that in the normal group (*p* < 0.05) (Figure 2B). Mice that received intragastric administrations of either low or high doses of probiotic compounds exhibited significantly shorter times to the first black stool defecation compared to the model group (*p* < 0.05). This result indicates that probiotic compounds can significantly improve the intestinal transit rate in constipation model mice by improving intestinal function and intestinal motility, thereby reducing the time till the first black stool defecation. However, there was no significant difference between the low and high-dose groups in reducing the time until the first black stool defecation. At the same time, the numbers and weights of black stools in the low and high-dose groups were significantly increased compared with the model group (*p* < 0.05), but there were no significant differences between the low and high-dose groups (Figure 2C,D).

### 3.2. Effects of Probiotic Compounds on Serum Levels of Gastrointestinal Regulatory Hormones in Mice with Constipation

**Figure 3:**
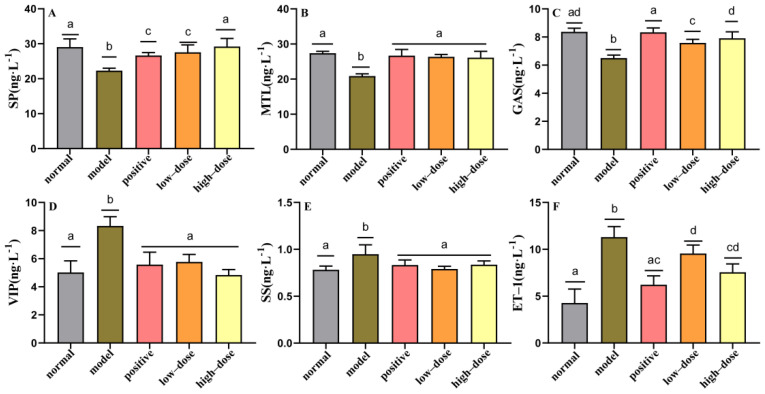
Effects of probiotic compounds on gastrointestinal peptides in the sera of constipated mice. (**A**) Substance P (SP); (**B**) motilin (MTL); (**C**) gastrin (GAS); (**D**) vasoactive intestinal peptide (VIP); (**E**) somatostatin (SS); (**F**) endothelin (ET–1). Data are means with SEM. a–d: Different letters indicate significant differences (*p* <0.05), and the same letters or no letters indicate no significant difference.

To investigate the effects of probiotic compounds on adhesion properties and tolerance of gastric and small intestine juices, the serum levels of gastrointestinal peptide neurotransmitter MTL, GAS, SP, ET-1, SS, and VIP were evaluated in the serum samples of mice with constipation. After the loperamide challenge, the serum concentrations of SP, MTL, and GAS were significantly reduced in model mice compared to the normal group (*p* < 0.05) (Figure 3A–C); and the concentrations of VIP, SS, and ET-1 were significantly increased in model mice relative to the control group (*p* < 0.05) (Figure 3D–F), suggesting that loperamide-induced constipation may also be associated with abnormalities in serum SP, MTL, GAS, VIP, SS, and ET-1 concentrations. The concentrations of all regulatory hormones showed a recovery trend in the sera of mice treated with probiotic compounds (Figure 3). We found that the serum concentrations of MTL, VIP, and SS could be restored to their normal levels in all groups by the administration of either high or low doses of probiotic compounds. The high-dose administrations of probiotic compounds were able to restore the serum concentrations of SP and GAS to normal. Compared to the low dose, the high doses of probiotic compounds were better able to restore the concentrations of SP and GAS in mice with constipation. These probiotic strains may increase GI transit in mice with constipation by regulating the secretion of gastrointestinal regulatory hormones in a dose-dependent manner.

### 3.3. Effects of Probiotic Compounds on Intestinal Gastrointestinal Regulatory Hormones in Mice with Constipation

**Figure 4:**
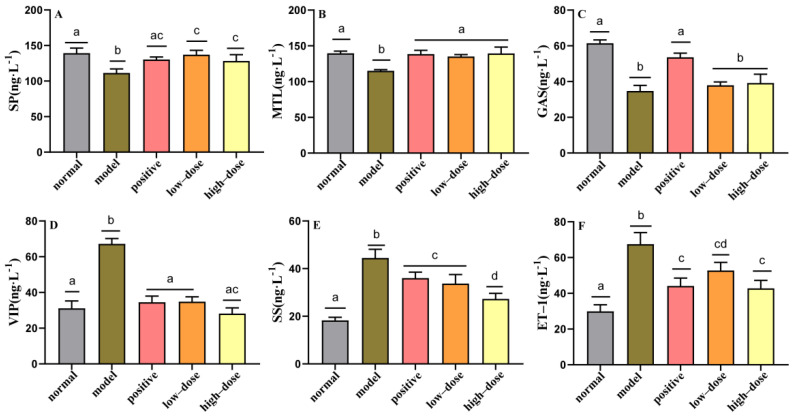
Effect of probiotic compounds on gastrointestinal peptides in intestinal tissues of constipated mice. (**A**) Substance P (SP). (**B**) Motilin (MTL). (**C**) Gastrin (GAS). (**D**) Vasoactive intestinal peptide (VIP). (**E**) Somatostatin (SS). (**F**) Endothelin (ET–1). Data are means with SEM. a–d: Different letters indicate significant differences (*p* < 0.05), and the same letters or no letters indicate no significant difference.

The loperamide challenge led to significant decreases in the levels of SP, MTL, and GAS in the colons of model mice relative to the normal group (*p* < 0.05) (Figure 4A–C). Additionally, the intestinal concentrations of VIP, SS, and ET-1 in model mice were significantly increased after the loperamide challenge (*p* < 0.05) (Figure 4D–F), suggesting that loperamide-induced constipation may also be associated with abnormalities in intestinal SP, MTL, GAS, VIP, SS, and ET-1 concentrations. Once again, the concentrations of all regulatory hormones showed a recovery trend in the sera of mice treated with probiotic compounds (Figure 4). The intestinal concentrations of MTL and VIP could be restored to their normal levels by the administration of either high or low doses of probiotic compounds. Taken together, these results indicate that probiotic compounds could restore the deficient levels of SP and MTL, and reduce the elevated levels of VIP, SS, and ET-1, thereby regulating gastrointestinal regulatory hormones and relieving constipation.

### 3.4. Effects of Probiotic Compounds on Serum Cytokines in Mice with Constipation

We found that treatments with probiotic compounds had no negative impacts on host health and did not destroy the cytokine levels in the host (Supplementary Figures S1 and S2).

### 3.5. Effects of Probiotic Compounds on the Gut Microbiome

**Figure 5:**
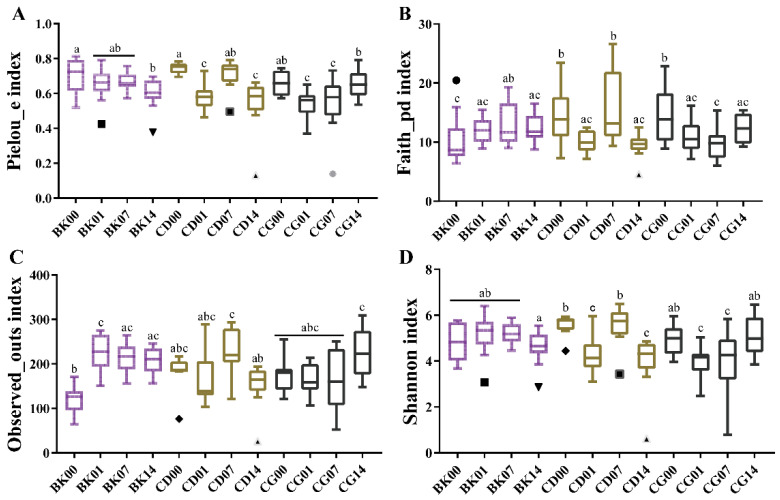
Treatment with probiotic compounds alters the diversity and structure of the gut microbiota. (**A**) α-Diversity index: Pielou_e. (**B**) α-Diversity index: Faith_pd. (**C**) α-Diversity index: Observed_otus. (**D**) α-Diversity index: Shannon. Normal group, BK; low-dose group, CD; high-dose group, CG; the first gavage, 00; the last gavage, 01; one week after stopping gavage, 07; two weeks after stopping gavage, 14. Statistical significance between groups is indicated by a–c, and different letters indicate that there was a significant difference between groups (*p* < 0.05, ANOVA, Tukey test).

The components of α diversity (Pielou evenness, Faith index, Observed_otus, and Shannon index) were calculated to illustrate the effects of different concentrations of probiotic compounds on the gut microbiota. These indices allowed us to assess the changes in species richness and evenness, along with the overall diversity of the gut microbiota, at different intervention times. After the first gavage, the last gavage, one week after stopping gavage, and two weeks after stopping gavage, the Pielou index and Shannon index of the intestinal microbiota in the BK group remained stable, and the Faith and Observed–otus did not change significantly. However, the Pielou, Faith index, Observed_otus, and Shannon index changed in the groups fed probiotic compounds (Figure 5). In addition, different doses of probiotic compounds affected the species richness of the intestinal microbiota of mice. After stopping gavage for two weeks, the Pielou_e and Shannon index in the CD group were lower than those after the first gavage (*p* < 0.05). The Faith_pd index changed significantly between one and two weeks after stopping gavage (*p* < 0.05), but there was no significant difference between these points in the CG group (Figure 5D). The above results indicate that probiotic compounds affected the abundance and homogeneity of gut microbiota relative to the BK group, and different concentrations of probiotic compounds had different effects on the mice’s microbiota over time after the end of the intervention. This may indicate that high-dose probiotic compounds maintain the diversity of intestinal microbiota and better maintain the stability of intestinal health. Thus, we conclude that probiotic compounds can colonize the intestines of mice, and different concentrations of probiotic compounds have different effects on colonization.

**Figure 6:**
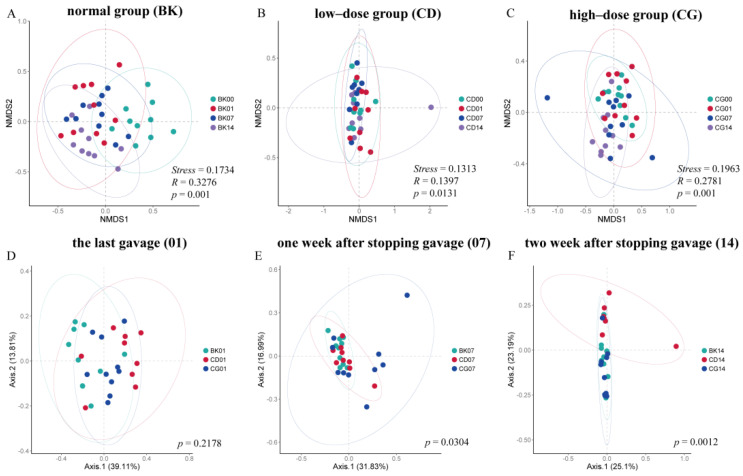
The effects of probiotic compounds on the overall structure of mouse gut microbiome. β-Diversity of gut microbiota at different time in (**A**) BK group, (**B**) CD group, (**C**) CG group. Principal component analysis based on Bray–Curtis distance matrices showed differences in the β-diversity of the gut microbiome among all groups at (**D**) the last gavage, (**E**) one week after stopping gavage, and (**F**) two weeks after stopping gavage. Normal group, BK; low-dose group, CD; high-dose group, CG; the first gavage, 00; the last gavage, 01; one week after stopping gavage, 07; two weeks after stopping gavage, 14.

Based on three different β-diversity distances (Bray–Curtis), the composition of gut microbiota was analyzed after the first gavage, the last gavage, one week after stopping gavage, and two weeks after stopping gavage with different concentrations of probiotic compounds. NMDS based on the Bray–Curtis index was used to analyze the beta diversity of gut microbiota in the four groups. The β-diversity showed that there was no significant change in the gut microbiota between the group fed probiotic compounds and the control group at the first gavage (Supplementary Figure S3), but the β-diversity of gut microbiota changed significantly over time (*p* < 0.05) (Figure 6A–C). Such findings indicate that the probiotic compounds affected the overall structure of the gut microbiota. After stopping gavage for two weeks, the gut microbiota in both the CD and CG groups still demonstrated a significant change (*p* < 0.05). PCoA analysis showed that the compositions of gut microbiota in the CD and CG groups changed significantly after the last gavage and two weeks after stopping gavage compared with the first gavage (Figure 6B,C). One week after stopping gavage and two weeks after stopping gavage, the microbiota in the CD and CG groups were different from that of the BK (*p* < 0.05) (Figure 6E,F).

The above results tell us that although the gut microbiota of each group did not change significantly at the beginning of the intervention, probiotic compounds were able to influence the gut microbiota, and that the effects persisted even when the interventions were stopped. This effect varied with time after the end of the intervention period. In addition, these results are consistent with the aforementioned results of the effects on intestinal microbiota diversity, further indicating that probiotic compounds were able to colonize the intestines of mice and that different concentrations of probiotic compounds had different effects on colonization.

**Figure 7:**
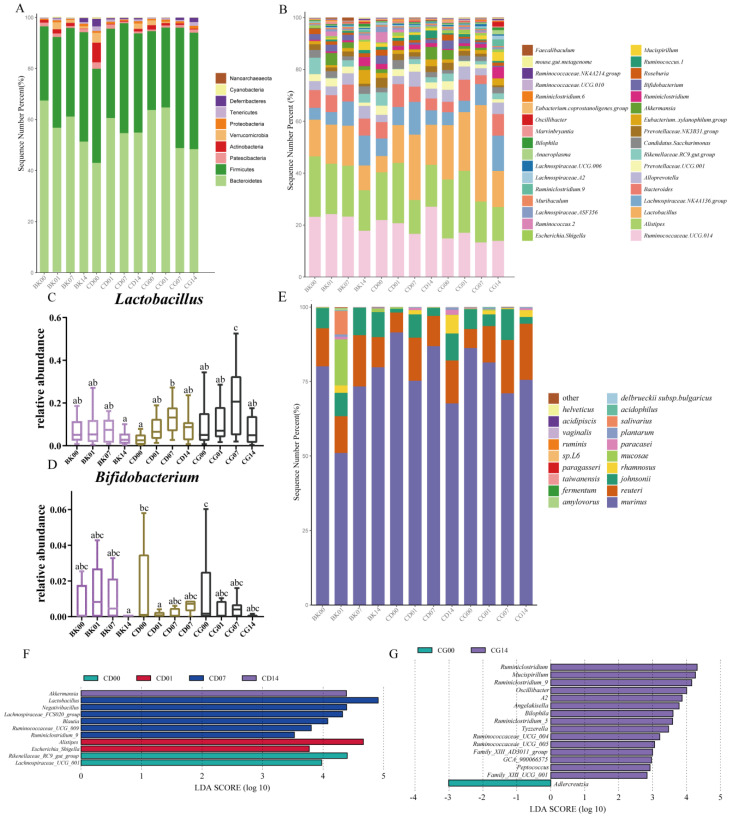
The effects of probiotic compounds on the composition of gut microbiota. (**A**) Microbial distribution at the phylum level. (**B**) Microbial distribution at the genus levels. (**C**,**D**) Relative abundances of the genera *Lactobacillus* and *Bifidobacterium*. Statistical significance between groups is indicated by a–c: different letters indicate that there is a difference between groups (*p* < 0.05, ANOVA, Tukey test). (**E**) Treatment with different concentrations of probiotic compounds altered the gut microbiota at the Lactobacillus species level. (**F**) Effects of low-dose probiotic compounds on the gut microbiota of mice at different times. (**G**) Effects of high-dose probiotic compounds on the gut microbiota of mice at different times. Normal group, BK; low-dose group, CD; high-dose group, CG; the first gavage, 00; the last gavage, 01; one week after stopping gavage, 07; two weeks after stopping gavage, 14.

At the phylum level, the gavage of probiotic compounds led to a decrease in the relative abundance of Bacteroides and an increase in that of Firmicutes (Figure 7A). A percentage accumulation map of the microbiota with the relative abundances of the top thirty at the genus level was created to reveal the gut microbial alterations. The abundances of *Lactobacillus* were high in the CD and CG groups, and *Akkermansia* was enriched in both CD14 and CG00 groups (Figure 7B). At the genus level, the relative abundance of *Lactobacillus* in the CD and CG groups increased with the gavage series, but slightly decreased with time after the end of the gavage period (Figure 7C). The relative abundance of *Bifidobacterium* decreased after the feeding of probiotic compounds, and did not change significantly after gavage stopped (Figure 7D). We further explored which *Lactobacillus* species changed after gavage, and found that *Ligilactobacillus murinus*, *Limosilactobacillus reuteri*, *Lactobacillus johnsonii*, and *Lacticaseibacillus rhamnosus* were enriched in CD and CG groups (Figure 7E). Lefse analysis was used to analyze the differences in gut microbiota of mice that were fed probiotic compounds at different times. In the CD group, the abundances of *Rikenellaceae* and *Lachnospiraceae* in fecal microbiota increased before gavage; the genes *Alistipes* increased significantly at the last gavage; the genera *Lactobacillus*, *Negativibacillus*, *Lachnospiraceae. FCS020.group*, *Blautia*, *Ruminococcaceae UCG_009*, and *Ruminiclostridium 9* increased significantly after one week of stopping gavage; and *Akkermansia* saw a significant increase two weeks after stopping gavage (Figure 7F). In contrast, in the CG group, the genera that significantly increased two weeks after stopping gavage included *Ruminiclostridium*, *Mucispirillum*, *Ruminiclostridium 9*, *Oscillibacter*, *Angelakisella*, *Bilophila*, *Ruminiclostridium 5*, *Tyzzerella*, *Ruminococcaceae UCG_004*, *Ruminococcaceae UCG_005*, *Ruminococcaceae UCG_003*, and *Peptococcus* (Figure 7G). The above results demonstrate that the gut microbiota of the mice were significantly altered at the genus level after interventions with probiotic compounds.

Different concentrations of probiotic compounds affected the intestinal microbiome diversity, and continued to regulate the gut microbiota after the intervention was stopped, indicating that this product may colonize the intestinal tracts of mice. Therefore, we performed absolute quantitative sequencing of *Lactobacillus* and *Bifidobacterium* to further determine the amounts of compound probiotic colonization in the mice’s intestines.

### 3.6. Colonization of Probiotic Compounds in Gut Microbiota

**Figure 8:**
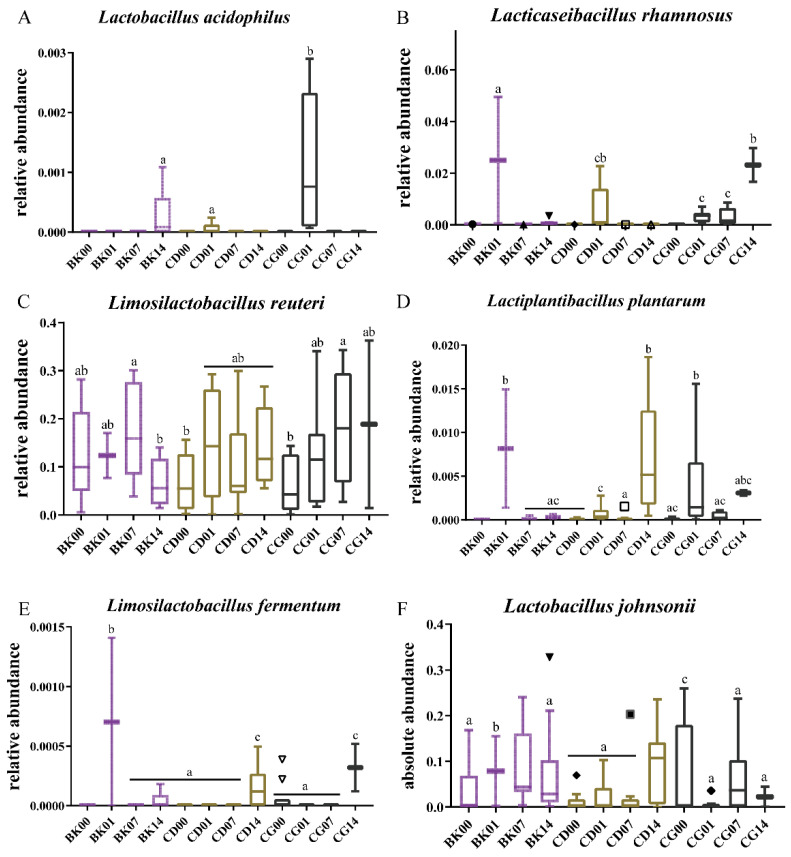
Effects of different probiotic compound combinations on the gut microbiota at the Lactobacillus species level. (**A**–**F**) Relative abundances of the genera *Lactobacillus acidophilus*, *Lacticaseibacillus rhamnosus*, *Limosilactobacillus reuteri*, *Lactiplantibacillus plantarum*, *Limosilactobacillus fermentum*, and *Lactobacillus johnsonii*. Normal group, BK; low-dose group, CD; high-dose group, CG; the first gavage, 00; the last gavage, 01; one week after stopping gavage, 07; two weeks after stopping gavage, 14. Statistical significance between groups is indicated by a–c: different letters indicate that there is a difference between groups (*p* < 0.05, ANOVA, Tukey test).

**Figure 9:**
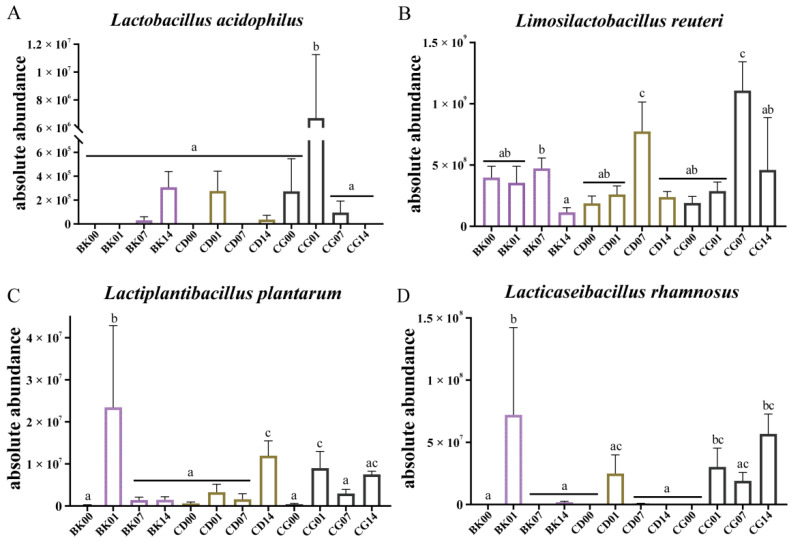
Absolute abundances of the Lactobacillus species with respect to the effects of probiotic compounds. Probiotic compounds included (**A**) *Lactobacillus acidophilus*; (**B**) *Limosilactobacillus reuteri*; (**C**) *Lactiplantibacillus plantarum*; (**D**) *Lacticaseibacillus rhamnosus*. Normal group, BK; low-dose group, CD; high-dose group, CG; the first gavage, 00; the last gavage, 01; one week after stopping gavage, 07; two weeks after stopping gavage, 14. Statistical significance between groups is indicated by a–c: different letters indicate that there is a difference between groups (*p* < 0.05, ANOVA, Tukey test).

At the first gavage, the gut microbiota in the BK, CD, and CG groups mainly contained *L. rhamnosus*, *L. reuteri*, and *L. johnsonii* (Figure 7E). The relative abundance of *L. acidophilus* in the mouse intestine increased after the last gavage with probiotic compounds (*p* < 0.05). The colonization effect of the CG group was higher than that of the CD group; however, its relative abundance gradually returned to the state before gavage as time progressed, which indicates that the *L. acidophilus* in probiotic compounds could not colonize the mouse intestine for a long time. The relative abundance of *L. acidophilus* in the intestines of mice in the CD and CG groups reached its peak during the last gavage (Figure 8A). Our findings highlight that different concentrations of probiotic compounds have different effects on *L. acidophilus* colonization in the mouse intestine. After calculating its absolute content, it was found that *L. acidophilus* increased only during the last gavage in the CG group (*p* < 0.05), and that there were no major fluctuations in any other group during other periods (Figure 9A). This would suggest that the probiotic compounds cannot promote long-term colonization of *L. acidophilus* in the intestine.

The relative contents of *L. rhamnosus* and *L. plantarum* in the intestinal tract of the BK group were almost zero. After the last gavage of probiotic compounds, the relative abundances of these two types of *Lactobacillus* increased slightly (Figure 8B,D). Two weeks after stopping gavage, the relative abundances of *L. plantarum* in both CD and CG groups increased (*p* < 0.05); the increase in CD was larger than in CG (*p* > 0.05) (Figure 8D). Plus, the relative abundance of *L. rhamnosus* in CG increased. This indicates that *L. plantarum* and *L. rhamnosus* can be colonized very well in the mouse intestine under the beneficial effects of probiotic compounds, and that the colonization of *L. rhamnosus* was better at high concentrations, whereas the colonization of *L. plantarum* was better at low concentrations. The absolute content calculation results also showed that *L. plantarum* and *L. rhamnosus* had no major fluctuations after the first gavage, end of gavage, or one week after stopping gavage; but when the time reached two weeks after gavage, the absolute contents of *L. plantarum* and *L. rhamnosus* increased much more in mice fed probiotic compounds compared to the BK group (Figure 9C,D). This demonstrated that *L. plantarum* and *L. rhamnosus* indeed did colonize in the intestine, and for *L. rhamnosus* the effect of a high concentration was much greater than that of a low concentration. The colonization of *L. plantarum* was better at low concentrations.

Regardless of the relative or absolute level of *L. reuteri*, the abundance of *L. reuteri* increased compared the first gavage (Figure 8C and Figure 9B). The effects of probiotic compounds on the *L. reuteri* colonization could be evaluated. The relative abundance of *L. reuteri* was increased gradually in CG groups, and the absolute contents increased in CD and CG groups, but decreased two weeks after stopping gavage compared to one weeks after stopping gavage. Such findings indicate that the probiotic compounds affected the structure of the gut microbiota, and *L. reuteri* could only colonize the intestines for a short time.

In addition to the changes in the relative abundances of *L. acidophilus*, *L. plantarum*, *L. reuteri*, and *L. rhamnosus*, the relative abundances of other microorganisms in the mouse intestine also changed after gavaging with different concentrations of probiotic compounds (Figure 8). The relative amounts of *L. fermentum* increased two weeks after stopping gavage in mice fed probiotic compounds. The relative abundance of *Lactobacillus johnsonii* increased two weeks after stopping gavage in CD group, and decreased two weeks after stopping gavage in the CG group. Importantly, such findings indicate that high doses of probiotics promoted the growth of *L. fermentum*, whereas low doses promoted that of *L. johnsonii* (Figure 8E,F). Through absolute quantification of *Bifidobacterium animalis* subsp. *Lactis*, it was found that the BK group had a high content of *B. lactic*, and the CD group had a high content of *B*. *lactis*, initially. After one month of intragastric administration, the content of *B. lactic* decreased, and the content of *B. lactic* gradually increased and remained stead, indicating that the *B. lactic* in the intestines have the ability to self-regulate. The *B. lactic* did not increase after gavage with high-dose probiotics compounds, and its content gradually decreased after the gavage, which indicated that the ability of *B. lactic* to colonize the intestinal tract in the high-dose group was not as good as in the low-dose group (Supplementary Figure S3).

## 4. Discussion

Constipation is a common and highly prevalent condition worldwide [18,19]. It is conventionally treated with laxatives and other drugs that cannot be used long-term because of their side effects [20]. Therefore, there is an urgent need for a safe and long-term treatment for constipation. To that end, the use of probiotics has been recommended by some scholars based on evidence from animal and clinical studies [8,21,22]. Despite the popularity of probiotics as a dietary approach for the treatment of constipation, their specific mechanism of action remains unknown [23]. In this study, the effects of probiotic compounds on the symptoms of constipation in mice and their mechanisms of colonization in gut microbiota were investigated. The probiotic compounds can promote bowel movement, improve bowel movement frequency, increase the number and weight of stools, and effectively alleviate constipation. We found that the symptoms of constipation and gut microbiome composition were both significantly changed as a result of interventions with probiotic compounds. Thus, our results provide valuable insights into the interactions among probiotics, the microbiome, and constipation.

Recent clinical studies have found that probiotics can be a useful tool for the treatment of constipation and can yield significant results [24,25,26], such as improvements in gastrointestinal regulatory peptides, neurotransmitters, neurotrophic factors, and the gut microbiota. We found that probiotic compounds could improve the GI transit rate significantly in low and high-dose groups by improving intestinal function and intestinal motility, thereby reducing the time till the first black stool defecation. Treatment with probiotic compounds can effectively improve intestinal motility and accelerate the movement and exit of chyme via the small intestine. The GI transit rate reflects the dynamics of the small intestine and may thus reflect the effect of probiotic compounds on intestinal motility. We found no significant difference between the low and high-dose groups in improving either the intestinal transit rate or small intestinal motility. The gastrointestinal hormones play important physiological roles in the regulation of the motor activity of the gastrointestinal tract. In our study, we found that probiotic compounds can enhance the abnormalities of SP, MTL, and GAS, and reduce the abnormalities of VIP, SS, and ET-1, thereby regulating gastrointestinal regulatory hormones and relieving constipation.

Intake of probiotics can regulate the fecal microbiota, increase the levels of organic acids to promote intestinal peristalsis, shorten the colon operation time, and lessen symptoms of constipation. Studies have also found that the abundances of *Bifidobacterium* and *Lactobacillus* in the feces of adults with constipation are significantly reduced [27,28]. After supplementing with specific probiotics, constipation can be improved by promoting intestinal peristalsis and defecation frequency under the action of the gut microbiome [29,30,31], mainly by increasing the relative abundance of *Ruminococcaceae* [32]. In a population experiment using the probiotics of *Streptococcus thermophilus* and *L. plantarum*, it was found that the abundances of *Lactobacillus* and *Bifidobacterium* in the intestines increased after the intervention, thereby shortening the transit time of feces in the intestine and increasing the frequency and volume of bowel movements [33]. In addition, prebiotic chitosan oligosaccharides can significantly increase intestinal motility, inhibit intestinal barrier damage and the inflammatory response, and improve the water–electrolyte balance in constipation model mice, thereby increasing the frequency of defecation and the dry-wet weight ratio of feces in mice [34]. They further explored why chitosan oligosaccharides can relieve constipation, and found that it improves the gut microbiome imbalance of constipated mice at the levels of phyla, family, and genus. They accomplish this by reducing the ratio of Firmicutes to Bacteroides [35]; increasing the abundances of *Bacteroides, Lactobacillus,* and *Faecalibaculus*; and decreasing the populations of *Bllophila*, *Lachnospiraceae,* and *Ruminococcaceae* in constipated mice, thereby regulating the metabolism of bile acid and tryptophan [34,36]. Tryptamine produced by the metabolism of tryptophan by gut bacteria can activate G-protein-coupled receptors (5-HT_4_ receptors) on colonic epithelial cells, thereby increasing colonic secretion, promoting gastrointestinal transport, and bringing inspiration for the treatment of constipation [12]. In our experiment, it was also found that *Lactobacillus* abundance increased after intervention with low and high doses of probiotic compounds; *Ruminococcaceae* abundance increased after intervention with low and high doses of probiotic compounds; and *Akkermansia* were enriched two weeks after stopping gavage in the low-dose group (Figure 6F). A double-blind, randomized, cross-over intervention study analyzed the effect of inulin consumption on stool frequency in healthy adults with mild constipation. They found inulin did not significantly change the metabolome and had a mild effect on the composition of the gut microbiota. It specifically increased the *Anaerostipes* and *Bifidobacterium* abundances, and reduced the relative abundance of *Bilophila* [37]. However, in our experiment, *Bilophila* had a significant role two weeks after stopping gavage in the CG group (Figure 7G). This difference could be due to differences in the intestinal microbiota of humans and mice. In addition, probiotics can be regarded as safe and natural agents for the alleviation of functional constipation in adults, and there are some probiotic products that can relieve constipation. The strains used included *Streptococcus salivarius* subsp. *thermophilus*, *Enterococcus faecium*, *L. rhamnosus* GG, *L. acidophilus*, *L. plantarum*, *Lacticaseibacillus paracasei*, *L. bulgaricus*, and *Bifidobacterium* (*breve* and *animalis* subsp. *lactis*) [38,39], and the weekly stool volumes of probiotic-treated subjects increased significantly. The probiotic compounds we used to gavage the mice also contained *L. acidophilus*, *L. rhamnosus*, *L. reuteri*, *L. plantarum*, and *Bifidobacterium animalis* subsp. *lactis*. More importantly, the probiotic compounds were found to modulate intestinal bacterial communities—the main gut microbiota in the CD and CG group became *L. rhamnosus*, *L. reuteri,* and *L. johnsonii*. Even two weeks after stopping the gavage, we found that *L. plantarum* and *L. rhamnosus* could still colonize the intestinal tract, and that a higher dose of probiotic could promote the colonization of specific strains. In addition, studies have reported that *L. plantarum* and *L. reuteri* can metabolize tryptophan and produce metabolites that help relieve constipation [40]. The above results show that different concentrations of probiotic compounds promote *Lactobacillus* colonize the intestines of mice, and that the colonization after high-dosage probiotic treatments was generally better than after low-dose treatments. From the perspective of the relative abundances of specific species, the colonization extents of different *Lactobacillus* species in the mouse intestine were also different. The colonization extents of *L. plantarum* and *L. rhamnosus* were generally better than those of *L. acidophilus* and *L. reuteri*. In addition, different concentrations of probiotic compounds impacted the species composition of *Lactobacillus* in the gut microbiota of the mice.

## 5. Conclusions

The probiotic compounds alleviated the symptoms of constipation, improved the rate of intestinal motility, and increased the amount of stool. At the same time, the *Lactobacillus* and *Bifidobacterium* in the intestinal tract were changed to regulate the gut microbiota. More importantly, after the intervention was over, the abundances of probiotic compounds could still be detected, which meant that they could be colonized in the intestinal tract and played a role in regulating the gut microbiota and alleviating constipation. The effect of regulating the microbiota of the five strains combined might be better than that of a single probiotic. We found that probiotic compounds are beneficial for the clinical treatment of constipation.

## Data Availability

All data generated or used during the study appear in the submitted article. The raw sequencing data have been deposited in the NCBI SRA repository, bioproject accession number is PRJNA793720.

## Supplementary Materials

Figure S1: Effect of probiotic compounds on serum cytokines in a mouse model of constipation, (A)IL-2. (B) IL- 4. (C) IL-6. (D) IL-10. (E) IL-12. (F) IL-17. (G) TNF-α. (H) TNF-γ. Figure S2: Effect of probiotic compounds on serum indexes in a mouse model of constipation. (A) Occludin. (B) D-lactic acid. (C) sIgA. Figure S3: Principal component analysis based on Bray–Curtis distance matrices showed difference in the β-diversity of the gut microbiome at the first gavage and absolute abundance of the Bifidobacterium animalis subsp. lactis. Table S1: Bacterias 16S rRNA V3-V4 50μL PCR reaction system.

## Sample Availability

Not available.
