# Supplementary material for: Efficacy of Probiotic Compounds in Relieving Constipation and Their Colonization in Gut Microbiota
Source: Molecules. 2022 Jan 20;27(3):666. doi: 10.3390/molecules27030666 (PMC8838973; doi:10.3390/molecules27030666)
Supplement: Supplementary file 1 [file molecules-27-00666-s001.zip › molecules-1540138-supplementary.pdf]

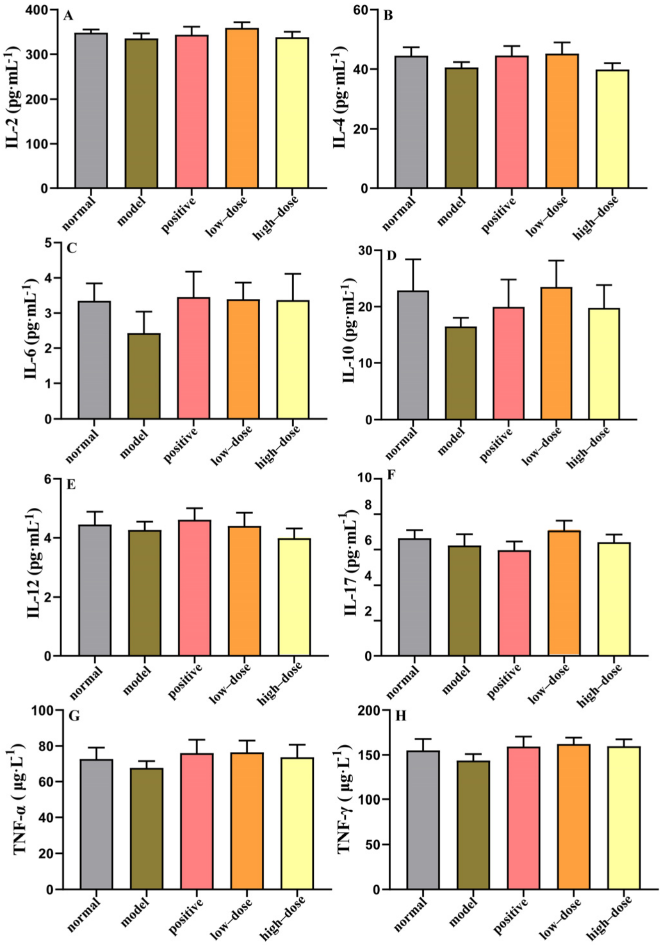

Figure S1. Effect of probiotic compounds on serum cytokines in a mouse model of constipation, (A)IL-2. (B) IL- 4. (C) IL-6. (D) IL-10. (E) IL-12. (F) IL-17. (G) TNF-α. (H) TNF-γ. Data are means with SEM; a-d: Different letters indicate significant differences (P<0.05), and the same letters and no letters have no difference significance.

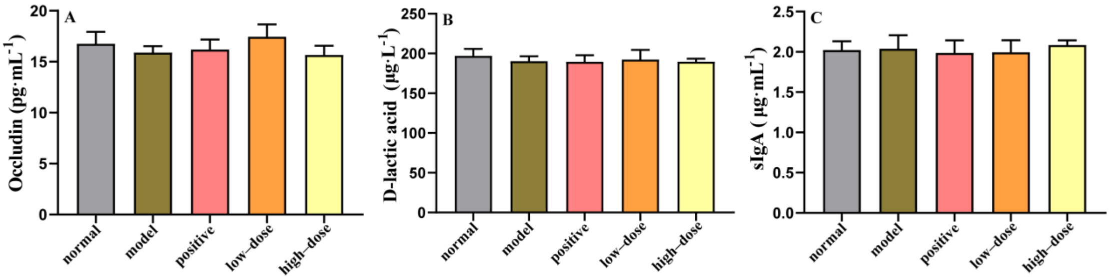

Figure S2. Effect of probiotic compounds on serum indexes in a mouse model of constipation. (A) Occludin. (B) D-lactic acid. (C) sIgA. Data are means with SEM; a-d: Different letters indicate significant differences (P<0.05), and the same letters and no letters have no difference significance.

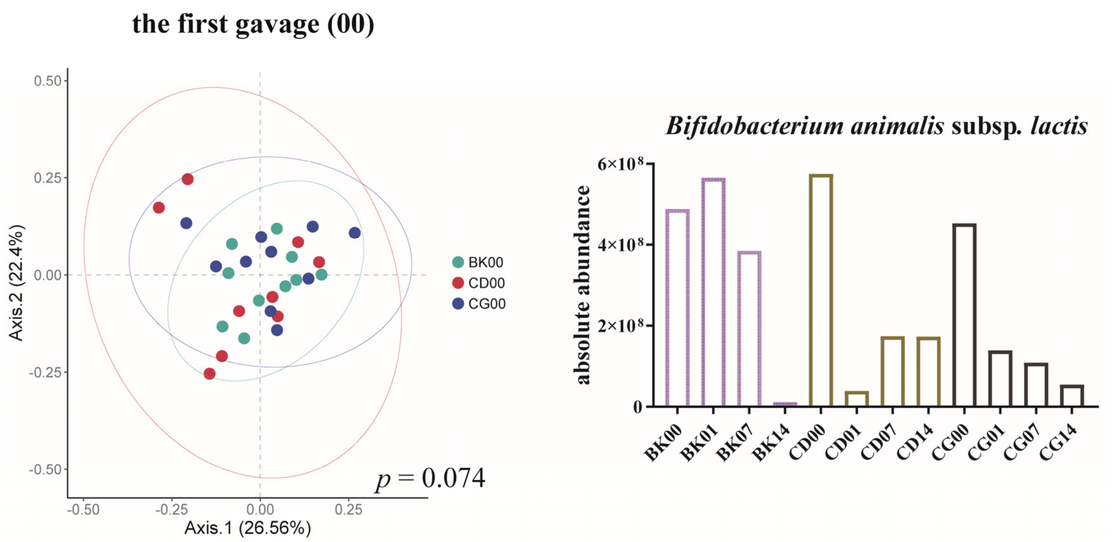

Figure S3. Principal component analysis based on Bray-Curtis distance matrices showed difference in the β-diversity of the gut microbiome at the first gavage and the absolute abundance of the Bifidobacterium animalis subsp. lactis.

Component	Amount（μL）
Taq mix	25
341F	1.5
806R	1.5
DNA	2
dd H_2O	20

Table S1: Bacterias 16S rRNA V3-V4 50 μL PCR reaction system. PCR condition: 95℃ 5 min, 95 ℃ 30 s, 52 ℃ 30 s, 72 ℃ 30s, 30 cycles, 72 ℃ 10 min, 12℃ 10 min.
